# Difluoro­[2-(quinolin-2-yl)phenolato]borane

**DOI:** 10.1107/S1600536811011895

**Published:** 2011-04-07

**Authors:** Xi Yang, Min Xia

**Affiliations:** a Department of Chemistry, Zhejiang Sci-Tech University, Hangzhou 310018, People’s Republic of China

## Abstract

The title compound, C_15_H_10_BF_2_NO, was synthesized by the reaction of 2-(quinolin-2-yl)phenol and boron trifluoride etherate. The quinoline ring system and the benzene ring are twisted, making a dihedral angle of 8.3 (2)°. In the crystal, π–π inter­actions between the aromatic rings [centroid–centroid distance = 3.638 (9) Å] link the mol­ecules into chains propagating in [100].

## Related literature

For the properties and the preparation of difluoro­boron complexes, see: Loudet *et al.* (2007 ▶); Ulrich *et al.* (2008 ▶); Ono *et al.* (2009 ▶); Zhou *et al.* (2008 ▶); Xia *et al.* (2008 ▶).
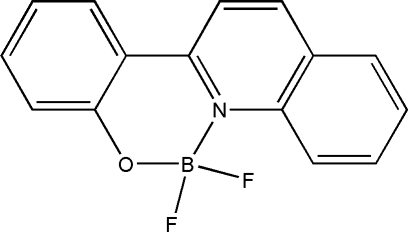


## Experimental

### 

#### Crystal data

C_15_H_10_BF_2_NO
*M* *_r_* = 269.05
Triclinic, 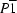

*a* = 7.4660 (15) Å
*b* = 8.6300 (17) Å
*c* = 9.3420 (19) Å
α = 97.71 (3)°
β = 95.63 (3)°
γ = 92.61 (3)°
*V* = 592.5 (2) Å^3^
*Z* = 2
Mo *K*α radiation
μ = 0.12 mm^−1^
*T* = 295 K
0.46 × 0.22 × 0.14 mm

#### Data collection

Rigaku R-AXIS RAPID diffractometer
Absorption correction: multi-scan (*ABSCOR*; Higashi, 1995 ▶) *T*_min_ = 0.949, *T*_max_ = 0.984
4885 measured reflections
2169 independent reflections
1329 reflections with *I* > 2σ(*I*)
*R*_int_ = 0.025

#### Refinement

*R*[*F* ^2^ > 2σ(*F* ^2^)] = 0.040
*wR*(*F* ^2^) = 0.127
*S* = 1.13
2169 reflections
182 parameters
H-atom parameters constrained
Δρ_max_ = 0.29 e Å^−3^
Δρ_min_ = −0.20 e Å^−3^

### 

Data collection: *RAPID-AUTO* (Rigaku, 1998 ▶); cell refinement: *RAPID-AUTO*; data reduction: *CrystalStructure* (Rigaku/MSC, 2002 ▶); program(s) used to solve structure: *SHELXS97* (Sheldrick, 2008 ▶); program(s) used to refine structure: *SHELXL97* (Sheldrick, 2008 ▶); molecular graphics: *ORTEP-3* (Farrugia, 1997 ▶); software used to prepare material for publication: *SHELXL97* and *PLATON* (Spek, 2009 ▶).

## Supplementary Material

Crystal structure: contains datablocks global, I. DOI: 10.1107/S1600536811011895/si2346sup1.cif

Structure factors: contains datablocks I. DOI: 10.1107/S1600536811011895/si2346Isup2.hkl

Additional supplementary materials:  crystallographic information; 3D view; checkCIF report

## Figures and Tables

**Fig. 1.**
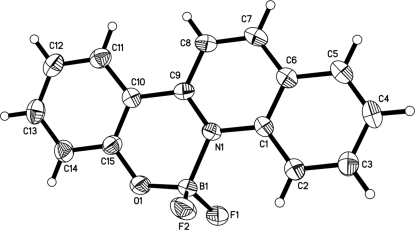
View of the title molecule. Displacement ellipsoids are drawn at the 30% probability level. H atoms are represented by circles of arbitrary size.

## supplementary crystallographic information

### Comment

Recently, the interest in synthesis and property research on novel difluoroboron complexes has been becoming increasingly intensive, due to their distinguishing fluorescence and important applications in chemical and biological fields [Loudet *et al.*, 2007]. Among them, the two types with N, N– and O, O-double dentate ligands are dominantly focused, like boradipyrromethene [Ulrich *et al.*, 2008] and 1, 3, 2- dioxaborine [Ono *et al.*, 2009] as the corresponding representatives. However, the isosteric analogues with N, O-double dentate ligands are limitedly reported, especially those having strong fluorescence intensity and high quantum yields [Zhou *et al.*, 2008]. We reported our example of N, O-double dentate difluoroborane complexes with outstandingly intensive green fluorescence based on 1,3-enamino-ketone structures [Xia *et al.*, 2008]. In connection with our study, herein we describe another complex exhibiting strong cyan fluorescence.

The bond lengths and angles of the title molecule (Fig. 1) are within normal ranges. The aromatic quinoline [C1–C9/N1] and benzene [C10–C15] rings are twisted at a dihedral angle of 8.3 (2)°. In the crystal structure, π-π interactions between the aromatic rings [Cg1···Cg2^1^ = 3.638 (9) Å, symm. code i = -*x*, -*y*, 1 - *z*] link molecules into chains propagated in direction [1 0 0], the two aromatic planes are partial overlap. The van der Waals forces stabilize further the crystal packing.

### Experimental

At room temperature, triethylamine (21 mmol, 2.9 mL) is added to the solution of 2-quinolin-2-yl- phenol (10 mmol, 2.21 g) in benzene(15 mL), the resulted mixture is stirred for 20 min and boron trifluoride etherate (30 mmol, 2.8 mL) is dropped into it. The large amount of yellow solid is precipitated after stirring for about 40 min, the solid is collected by filtration and washed by ether for several times. After dried in air, the corresponding difluoroboron complex is obtained in 92% yield as bright yellow powder(m.p. 537–538 K). At room temperature, ether is carefully and slowly dropped into the solution of the complex in dichoromethane and the resulted mixture is kept without any disturb under the airproof condition until the crystal is formed.

### Refinement

The structures were solved by Direct methods and using Fourier techniques. The non-hydrogen atoms were refined anisotropically. All H-atoms were placed in idealized locations with C–H distances 0.93 Å and refined as riding with appropriate thermal displacement coefficients *U*_iso_(H) = 1.2 times *U*_eq_(C).

### Crystal data

**Table:**

C_15_H_10_BF_2_NO	*Z* = 2
*M**_r_* = 269.05	*F*(000) = 276
Triclinic, *P*1	*D*_x_ = 1.508 Mg m^−^^3^
Hall symbol: -P 1	Mo *K*α radiation, λ = 0.71073 Å
*a* = 7.4660 (15) Å	Cell parameters from 3364 reflections
*b* = 8.6300 (17) Å	θ = 3.0–27.4°
*c* = 9.3420 (19) Å	µ = 0.12 mm^−^^1^
α = 97.71 (3)°	*T* = 295 K
β = 95.63 (3)°	Prism, yellow
γ = 92.61 (3)°	0.46 × 0.22 × 0.14 mm
*V* = 592.5 (2) Å^3^	

### Data collection

**Table:**

Rigaku R-AXIS RAPID diffractometer	2169 independent reflections
Radiation source: fine-focus sealed tube	1329 reflections with *I* > 2σ(*I*)
graphite	*R*_int_ = 0.025
Detector resolution: 10.00 pixels mm^-1^	θ_max_ = 25.4°, θ_min_ = 3.0°
ω scans	*h* = −8→9
Absorption correction: multi-scan (*ABSCOR*; Higashi, 1995)	*k* = −10→10
*T*_min_ = 0.949, *T*_max_ = 0.984	*l* = −11→11
4885 measured reflections	

### Refinement

**Table:**

Refinement on *F*^2^	Secondary atom site location: difference Fourier map
Least-squares matrix: full	Hydrogen site location: inferred from neighbouring sites
*R*[*F*^2^ > 2σ(*F*^2^)] = 0.040	H-atom parameters constrained
*wR*(*F*^2^) = 0.127	*w* = 1/[σ^2^(*F*_o_^2^) + (0.0631*P*)^2^] where *P* = (*F*_o_^2^ + 2*F*_c_^2^)/3
*S* = 1.13	(Δ/σ)_max_ < 0.001
2169 reflections	Δρ_max_ = 0.29 e Å^−^^3^
182 parameters	Δρ_min_ = −0.20 e Å^−^^3^
0 restraints	Extinction correction: *SHELXL*, Fc^*^=kFc[1+0.001xFc^2^λ^3^/sin(2θ)]^-1/4^
Primary atom site location: structure-invariant direct methods	Extinction coefficient: 0.033 (7)

### Special details

**Table:**

Geometry. All e.s.d.'s (except the e.s.d. in the dihedral angle between two l.s. planes) are estimated using the full covariance matrix. The cell e.s.d.'s are taken into account individually in the estimation of e.s.d.'s in distances, angles and torsion angles; correlations between e.s.d.'s in cell parameters are only used when they are defined by crystal symmetry. An approximate (isotropic) treatment of cell e.s.d.'s is used for estimating e.s.d.'s involving l.s. planes.
Refinement. Refinement of *F*^2^ against ALL reflections. The weighted *R*-factor *wR* and goodness of fit *S* are based on *F*^2^, conventional *R*-factors *R* are based on *F*, with *F* set to zero for negative *F*^2^. The threshold expression of *F*^2^ > σ(*F*^2^) is used only for calculating *R*-factors(gt) *etc*. and is not relevant to the choice of reflections for refinement. *R*-factors based on *F*^2^ are statistically about twice as large as those based on *F*, and *R*- factors based on ALL data will be even larger.

### Fractional atomic coordinates and isotropic or equivalent isotropic displacement parameters (Å^2^)

**Table:**

	*x*	*y*	*z*	*U*_iso_*/*U*_eq_	
F1	0.38077 (17)	0.27217 (11)	0.25453 (14)	0.0696 (4)	
F2	0.07658 (16)	0.22181 (12)	0.23344 (13)	0.0700 (4)	
O1	0.2311 (2)	0.34773 (14)	0.44802 (16)	0.0710 (5)	
N1	0.26036 (19)	0.06102 (14)	0.37319 (17)	0.0427 (4)	
C1	0.3005 (2)	−0.06216 (19)	0.2687 (2)	0.0441 (5)	
C2	0.3067 (3)	−0.0422 (2)	0.1235 (2)	0.0579 (6)	
H2	0.2826	0.0541	0.0939	0.069*	
C3	0.3479 (3)	−0.1635 (2)	0.0245 (2)	0.0640 (6)	
H3	0.3524	−0.1481	−0.0718	0.077*	
C4	0.3833 (3)	−0.3099 (2)	0.0649 (3)	0.0624 (6)	
H4	0.4125	−0.3905	−0.0037	0.075*	
C5	0.3750 (3)	−0.3344 (2)	0.2044 (3)	0.0563 (6)	
H5	0.3972	−0.4322	0.2313	0.068*	
C6	0.3324 (2)	−0.21077 (19)	0.3095 (2)	0.0465 (5)	
C7	0.3186 (3)	−0.2317 (2)	0.4545 (2)	0.0554 (5)	
H7	0.3407	−0.3284	0.4840	0.066*	
C8	0.2735 (3)	−0.1123 (2)	0.5524 (2)	0.0511 (5)	
H8	0.2625	−0.1285	0.6476	0.061*	
C9	0.2435 (2)	0.03633 (18)	0.5099 (2)	0.0425 (4)	
C10	0.1944 (2)	0.16571 (19)	0.6148 (2)	0.0441 (5)	
C11	0.1542 (3)	0.1444 (2)	0.7542 (2)	0.0556 (5)	
H11	0.1573	0.0449	0.7817	0.067*	
C12	0.1102 (3)	0.2672 (3)	0.8516 (2)	0.0629 (6)	
H12	0.0857	0.2506	0.9442	0.075*	
C13	0.1024 (3)	0.4155 (2)	0.8112 (2)	0.0602 (6)	
H13	0.0710	0.4983	0.8766	0.072*	
C14	0.1407 (3)	0.4412 (2)	0.6757 (2)	0.0591 (6)	
H14	0.1355	0.5411	0.6493	0.071*	
C15	0.1874 (3)	0.3172 (2)	0.5773 (2)	0.0498 (5)	
B1	0.2359 (3)	0.2321 (2)	0.3236 (3)	0.0502 (6)	

### Atomic displacement parameters (Å^2^)

**Table:**

	*U*^11^	*U*^22^	*U*^33^	*U*^12^	*U*^13^	*U*^23^
F1	0.0908 (10)	0.0476 (6)	0.0785 (9)	0.0031 (5)	0.0333 (7)	0.0210 (6)
F2	0.0803 (9)	0.0602 (7)	0.0707 (9)	0.0220 (6)	−0.0066 (7)	0.0181 (6)
O1	0.1229 (14)	0.0395 (7)	0.0548 (10)	0.0104 (7)	0.0259 (9)	0.0080 (6)
N1	0.0465 (9)	0.0372 (8)	0.0449 (10)	0.0025 (6)	0.0032 (7)	0.0089 (7)
C1	0.0420 (10)	0.0382 (9)	0.0508 (13)	0.0034 (7)	0.0027 (9)	0.0031 (8)
C2	0.0719 (14)	0.0489 (10)	0.0536 (14)	0.0113 (9)	0.0089 (11)	0.0057 (9)
C3	0.0769 (16)	0.0611 (12)	0.0542 (14)	0.0151 (10)	0.0107 (11)	0.0019 (10)
C4	0.0588 (13)	0.0511 (11)	0.0718 (17)	0.0090 (9)	0.0043 (11)	−0.0112 (11)
C5	0.0515 (12)	0.0393 (10)	0.0760 (16)	0.0048 (8)	0.0003 (11)	0.0044 (10)
C6	0.0384 (10)	0.0430 (10)	0.0567 (13)	−0.0010 (7)	−0.0011 (9)	0.0072 (8)
C7	0.0554 (12)	0.0402 (10)	0.0724 (15)	0.0034 (8)	0.0011 (11)	0.0179 (9)
C8	0.0569 (12)	0.0468 (10)	0.0516 (12)	0.0029 (8)	0.0018 (9)	0.0175 (9)
C9	0.0390 (10)	0.0449 (9)	0.0442 (12)	−0.0019 (7)	0.0006 (8)	0.0132 (8)
C10	0.0379 (10)	0.0442 (9)	0.0481 (12)	−0.0002 (7)	0.0004 (8)	0.0030 (8)
C11	0.0510 (12)	0.0656 (12)	0.0522 (13)	0.0037 (9)	0.0050 (10)	0.0158 (10)
C12	0.0570 (13)	0.0839 (15)	0.0485 (13)	0.0044 (10)	0.0110 (10)	0.0080 (11)
C13	0.0529 (13)	0.0657 (13)	0.0570 (15)	−0.0005 (9)	0.0095 (10)	−0.0106 (11)
C14	0.0676 (14)	0.0479 (10)	0.0603 (15)	0.0033 (9)	0.0124 (11)	−0.0023 (10)
C15	0.0556 (12)	0.0499 (11)	0.0440 (12)	−0.0009 (8)	0.0077 (10)	0.0065 (9)
B1	0.0666 (15)	0.0391 (11)	0.0489 (14)	0.0099 (9)	0.0138 (12)	0.0133 (9)

### Geometric parameters (Å, °)

**Table:**

F1—B1	1.367 (3)	C6—C7	1.403 (3)
F2—B1	1.381 (3)	C7—C8	1.360 (3)
O1—C15	1.338 (2)	C7—H7	0.9300
O1—B1	1.431 (3)	C8—C9	1.414 (2)
N1—C9	1.339 (2)	C8—H8	0.9300
N1—C1	1.408 (2)	C9—C10	1.468 (3)
N1—B1	1.619 (2)	C10—C11	1.398 (3)
C1—C2	1.395 (3)	C10—C15	1.400 (3)
C1—C6	1.410 (2)	C11—C12	1.374 (3)
C2—C3	1.368 (2)	C11—H11	0.9300
C2—H2	0.9300	C12—C13	1.384 (3)
C3—C4	1.396 (3)	C12—H12	0.9300
C3—H3	0.9300	C13—C14	1.369 (3)
C4—C5	1.354 (3)	C13—H13	0.9300
C4—H4	0.9300	C14—C15	1.394 (3)
C5—C6	1.420 (3)	C14—H14	0.9300
C5—H5	0.9300		
			
Cg1···Cg2^i^	3.638 (9)		
			
C15—O1—B1	124.65 (15)	N1—C9—C8	120.27 (16)
C9—N1—C1	120.50 (15)	N1—C9—C10	119.15 (16)
C9—N1—B1	121.28 (15)	C8—C9—C10	120.58 (18)
C1—N1—B1	118.22 (15)	C11—C10—C15	117.48 (17)
C2—C1—N1	121.58 (16)	C11—C10—C9	122.35 (17)
C2—C1—C6	118.53 (16)	C15—C10—C9	120.17 (18)
N1—C1—C6	119.88 (18)	C12—C11—C10	121.6 (2)
C3—C2—C1	120.35 (18)	C12—C11—H11	119.2
C3—C2—H2	119.8	C10—C11—H11	119.2
C1—C2—H2	119.8	C11—C12—C13	119.7 (2)
C2—C3—C4	121.3 (2)	C11—C12—H12	120.1
C2—C3—H3	119.3	C13—C12—H12	120.1
C4—C3—H3	119.3	C14—C13—C12	120.48 (18)
C5—C4—C3	119.90 (18)	C14—C13—H13	119.8
C5—C4—H4	120.1	C12—C13—H13	119.8
C3—C4—H4	120.1	C13—C14—C15	119.88 (19)
C4—C5—C6	120.01 (18)	C13—C14—H14	120.1
C4—C5—H5	120.0	C15—C14—H14	120.1
C6—C5—H5	120.0	O1—C15—C14	118.28 (17)
C7—C6—C1	118.21 (17)	O1—C15—C10	120.85 (16)
C7—C6—C5	121.96 (18)	C14—C15—C10	120.83 (19)
C1—C6—C5	119.83 (19)	F1—B1—F2	111.96 (18)
C8—C7—C6	120.81 (17)	F1—B1—O1	107.42 (17)
C8—C7—H7	119.6	F2—B1—O1	111.11 (16)
C6—C7—H7	119.6	F1—B1—N1	109.25 (15)
C7—C8—C9	120.25 (19)	F2—B1—N1	106.83 (15)
C7—C8—H8	119.9	O1—B1—N1	110.27 (17)
C9—C8—H8	119.9		

Symmetry codes: (i) −*x*, −*y*, −*z*+1.
